# Characterization of recombinant human lactoferrin *N*-glycans expressed in the milk of transgenic cows

**DOI:** 10.1371/journal.pone.0171477

**Published:** 2017-02-07

**Authors:** Annabelle Le Parc, Sercan Karav, Camille Rouquié, Elizabeth A. Maga, Apichaya Bunyatratchata, Daniela Barile

**Affiliations:** 1 Department of Food Science and Technology, University of California Davis, Davis, California, United States of America; 2 Department of Molecular Biology and Genetics, Canakkale 18 Mart University, Canakkale, Turkey; 3 Department of Animal Science, University of California Davis, Davis, California, United States of America; 4 Foods for Health Institute, University of California Davis, Davis, California, United States of America; University of Patras, GREECE

## Abstract

Lactoferrin (LF) is one of the most abundant bioactive glycoproteins in human milk. Glycans attached through *N-*glycosidic bonds may contribute to Lactoferrin functional activities. In contrast, LF is present in trace amounts in bovine milk. Efforts to increase LF concentration in bovine milk led to alternative approaches using transgenic cows to express human lactoferrin (hLF). This study investigated and compared *N*-glycans in recombinant human lactoferrin (rhLF), bovine lactoferrin (bLF) and human lactoferrin by Nano-LC-Chip-Q-TOF Mass Spectrometry. The results revealed a high diversity of *N-*glycan structures, including fucosylated and sialylated complex glycans that may contribute additional bioactivities. rhLF, bLF and hLF had 23, 27 and 18 *N-*glycans respectively with 8 *N*-glycan in common overall. rhLF shared 16 *N*-glycan with bLF and 9 *N*-glycan with hLF while bLF shared 10 *N*-glycan with hLF. Based on the relative abundances of *N*-glycan types, rhLF and hLF appeared to contain mostly neutral complex/hybrid *N*-glycans (81% and 52% of the total respectively) whereas bLF was characterized by high mannose glycans (65%). Interestingly, the majority of hLF *N*-glycans were fucosylated (88%), whereas bLF and rhLF had only 9% and 20% fucosylation, respectively. Overall, this study suggests that rhLF *N*-glycans share more similarities to bLF than hLF.

## Introduction

Milk is a source of lipids, minerals, carbohydrates and proteins for growth and development of the newborn. Besides all the nutrients necessary for the development of the newborn, human milk also provides protection against infection and inflammation and contributes to immune system and healthy microbial colonization [1]. There is extensive research on major compounds in milk, such as fats, proteins, carbohydrates and minerals, to understand their contribution to the biological roles of milks [2, 3]. Besides these compounds, milk also contains oligosaccharides that can be found free or attached to fats or proteins forming conjugated glycans. These glycans have interesting biological functions. They are involved in cell-to-cell and cell-to-microbe interactions, proper protein folding, stabilization, structural support and protective mechanisms, including the establishment of a protective intestinal flora in infants [4]. Free human milk oligosaccharides (HMOs), as well as the conjugated glycans, selectively stimulate the growth of a key infant gut microbe, *Bifidobacterium longum* subsp. *infantis* (*B*. *infantis*) that has various health benefits, including prevention of pathogen binding, positive modulation of intestinal epithelial cell responses and immune modulation [5–10].

Protein glycosylation, a post-translational modification of proteins with the attachment of sugar moieties, plays important roles in the structural conformation and bioactivity of proteins, including adhesion, targeting, folding and stability [11]. Glycans can be attached to the protein through *O*-glycosidic or *N-*glycosidic bonds. *N-*linked glycans (*N-*glycans) are linked via *N-*acetylglucosamines (HexNAc) to an asparagine residue of proteins in the particular amino acid sequence As*n-*X-Ser/Thr (where X can be any amino acid except proline) [12]. The *N-*glycan core is composed of two HexNAc and three mannose residues and is synthesized in the endoplasmic reticulum [12, 13]. Its elongation by other monosaccharides via the actions of glycosyltransferases and glycosidases determines the complexity and diversity of *N-*glycan structures [14]. *N-*glycans are classified by three main classes: high mannose, complex, or hybrid, depending on the modification of the core by various monosaccharides [12].

Lactoferrin (LF) is one of the most abundant glycoproteins in human milk [15]. It is an 80 kDa iron-binding glycoprotein that exhibits an array of biological activities, including antioxidant, antibacterial and antiviral activities, iron- (and other metal) binding and immunomodulation [16–18]. However, LF is present in very small amounts in bovine milk. The desire to develop infant milk formulas closer in composition to that of breast milk and the numerous possible applications of LF supplementation explain the growing interest in developing large-scale production methods of LF. Bovine LF (bLF) is used as a food and pharmaceutical supplement [19]. However, bLF doesn’t mimic all the biological roles of human LF (hLF) and its low concentration in bovine milk hinders the use of bovine milk as a LF source [20]. Several alternative approaches have emerged, including hLF production in transgenic organisms, for example, in rice and the milk of dairy cattle and goats [21, 22]. Because of its large capacity for milk production, the transgenic cow appears to be a cost-effective source for production of recombinant hLF (rhLF) on a large scale. rhLF appears to have similar physicochemical and biological properties as hLF, which has been corroborated by several *in vivo* studies [23].

Despite the various biological functions of lactoferrin, little is understood about its mechanism of action and the glycosylation pattern’s contribution to biological functions. In this present study, we compared *N-*glycan structures released from rhLF, bLF and hLF. Given the significant degree of homology between bLF and hLF (with 78% of shared sequence identity), the presence of similar glycan patterns was expected in the three proteins. Knowledge of detailed lactoferrin glycan patterns will be essential to understand the functionality of the protein.

## Materials and methods

### Milk and lactoferrin

Transgenic cows’ milk containing rhLF at 1.2 g/L was provided by Pharming Group NV’s herd in Wisconsin. Milk was collected from a transgenic Holstein in her second parity and stored at –20°C. The bovine milk retentate obtained by concentration of whey on a 10 kDa membrane, was provided by the University of California, Davis Milk Processing Laboratory. All the samples were stored at –20°C before use. hLF was purified from donor human milk by affinity chromatography as described by Barboza et al. [24].

### Lactoferrin purification

Prior to the purification of LF from transgenic milk, whey proteins were isolated from the other milk components using centrifugation and acidic precipitation. Milk was centrifuged at 4,000 × g for 30 min at 4°C to eliminate fat and part of the casein micelles. The aqueous phase between the upper fat layer and the casein pellet was collected. The pH of the collected fraction was decreased to pH 4.6 by addition of hydrochloric acid to precipitate the remaining casein micelles. The sample was centrifuged at 4,000 × g for 30 min at 4°C and the supernatant was collected. The centrifugation step was repeated and the supernatant was combined with the previous supernatant.

LF was purified from transgenic milk and bovine milk retentate by affinity chromatography as described by Le Parc et al. [25], with minor modifications. Affinity chromatography was performed by manually packing heparin Sepharose beads (GE Healthcare Life Sciences, Pittsburgh, PA, USA) into a 12-mL polypropylene column as a chromatographic support. The column was equilibrated with the running buffer (100 mM Tris pH 8, 0.05% Tween 20 and 0.05 M NaCl). Sample loading, washing and elution steps were performed manually. After loading the whey protein sample onto the column, the flow through was collected and reloaded on the column to increase LF-binding efficiency. This step was repeated two times. The sample was incubated with the heparin Sepharose beads for 3 h. The column was washed with running buffer to remove non-specifically bound proteins. The bound protein was eluted with a step-wise gradient using sodium chloride (NaCl) concentrations ranging from 0.1 to 1 M NaCl. Fractions were collected for each salt concentration and analyzed on 12% sodium dodecyl sulfate-polyacrylamide gel electrophoresis (SDS-PAGE) [26]. Fractions with higher LF concentration (without many other protein bands) were dialyzed (Spectra/Por^®^ 1 dialysis tubing, MWCO 6000–8000) against 20 mM sodium phosphate, pH 7.5 for 48 h.

A second step of purification using cation-exchange chromatography was performed to increase the purity of samples. LF was purified using a 1-mL pre-packed ion-exchange column (Bio-Scale Mini Macro-Prep High Q Cartridges, Bio-Rad, Hercules, CA, USA). All chromatographic steps were performed using an EP-1 model Bio-Rad Econo Pump and model 2110 Bio-Rad fraction collector at a 0.5 mL/min flow rate. The column was equilibrated with running buffer (20 mM sodium phosphate, pH 7.5) and samples were loaded onto the column. The flowthrough was collected and the column was washed with the running buffer. The bound protein was eluted with a step-wise gradient using NaCl concentrations ranging from 0.1 M to 0.7 M. The purity of LF fractions was evaluated by SDS-PAGE. Fractions with higher LF concentration were dialyzed with molecular porous membrane tubing (Spectra/Por ^®^ MWCO: 12,000–14,000) against water for 48 h. Protein concentrations in each sample were determined by the Bradford assay [27] using bovine serum albumin as the standard.

### *N-*glycan isolation

Purified bLF and rhLF in water were dried and reconstituted in 100 mM ammonium bicarbonate (NH_4_HCO_3_) (pH 8), 5 mM dithiothreitol (DTT). hLF was resuspended in 100 mM NH_4_HCO_3_ (pH 8), 5 mM DTT. Proteins were denatured by heating with four cycles alternating between boiling water (100°C) for 15 s and room temperature water for 2 min [28]. Two microliters of 500,000 units/mL peptidyl-*N*-glycosidase F (PNGase F; New England BioLabs, Ipswich, MA, USA) were added to the sample. The mixture was incubated overnight at 37°C under agitation. After enzymatic digestion, proteins were precipitated by the addition of 95% cold ethanol solution (4:1 ratio) to each sample and incubation at –20°C for 1 h. Samples were centrifuged at 4,000 × g for 30 min at 4°C. Pellets containing the precipitated proteins were discarded. Supernatants were collected and dried overnight by vacuum centrifugation. Samples were reconstituted in 600 μL of water.

Samples were loaded onto a porous graphitized carbon solid phase extraction (PGC SPE plate; Glygen, Columbia, MD, USA) that was conditioned using 3 x 100 μL of 80% ACN containing 0.1% TFA in water, followed by 3 x 100 μL of water. After sample loading, wells were washed using 6 x 200 μL of water and *N*-glycans were eluted using 3 x 200 μL of 40% ACN containing 0.1% TFA in water. The enriched *N*-glycans fraction was dried overnight under vacuum. Samples were rehydrated in 50 μL of water, mixed using a vortex mixer, and sonicated prior to mass spectrometry (MS) analysis. These samples were produced in triplicate.

### Analytical characterization of *N-*glycans

An Agilent 6520 accurate-mass Q-TOF LC/MS with a microfluidic nano-electrospray chip (Agilent Technologies, Santa Clara, CA, USA) was used to analyze N-glycan structures as described previously [29]. Briefly, two microliters of sample were loaded with solvent A (3% ACN, 0.1% formic acid in water (v/v)) at a capillary pump flow rate of 4 μL/min. *N-*glycan separation was performed on a 65-min gradient delivered by the nanopump at a flow rate of 0.3 μL/min. The 65-min gradient followed this program: 0% B (90% ACN, 0.1% formic acid in water (v/v) (0.0–2.5 min), 0 to 16% B (2.5–20.0 min), 16 to 44% B (20.0–30.0 min), 44 to 100% B (30.0–35.0 min) and 100% B (35.0–45.0 min). The mass range of 450–3000 m/z were used for *N-*glycans in the positive-ionization mode with an acquisition rate of 2.01 spectra/s. Mass calibration was performed with an internal calibrant ion of 922.010 m/z from the tuning mix (ESI-TOF Tuning Mix G1969−85000, Agilent Technologies). For tandem MS analysis of N-glycans, nitrogen gas was used to fragment the N-glycans structures within the mass range of 100–3000 m/z spectra. Acquisition was confirmed by MassHunter Workstation Data Acquisition software (Agilent Technologies).

N-glycan identification was performed with MassHunter Qualitative Analysis software (version B.04.00 SP2, Agilent Technologies) and the compounds were extracted using the Molecular Feature Extractor algorithm (chromatograms in a range of 400–3,000 m/z with a ≥1000 ion count cut-off, allowing charge states of +1–3, a retention time from 5–40 min). The extracted compounds were compared to bovine and human milk *N*-glycan libraries using a mass error tolerance of 20 ppm. *N*-glycans from the library included Hex, HexNAc, Fuc, NeuAc and NeuGc. The relative abundance of *N-*glycans was identified by MassHunter Profinder software (Agilent Technologies) using the Batch Targeted Feature Extraction algorithm (charge states of +1–3, mass error tolerance of 20 ppm and retention time tolerance of 1 min). The relative amount of each *N-*glycan was obtained by comparison with the total *N-*glycan area in each sample and the relative amount was expressed as a percentage of the total after the assignment of *N-*glycans was confirmed by tandem MS.

### Statistical analysis

To evaluate the significant differences (p ≤ 0.05) between different glycan structures released by each enzyme and type of whey protein one-way ANOVA (analysis of variance) was applied. Means of different groups were compared by Tukey’s multiple comparison test (Same letters means there is no significant difference between the groups). To visualize differences in the glycan structures released from each LF type, PCA (Principal Component Analysis) was employed. In addition, a heat map was created to specifically visualize the difference of glycan structures.

## Results

### Lactoferrin purification

**Fig 1:**
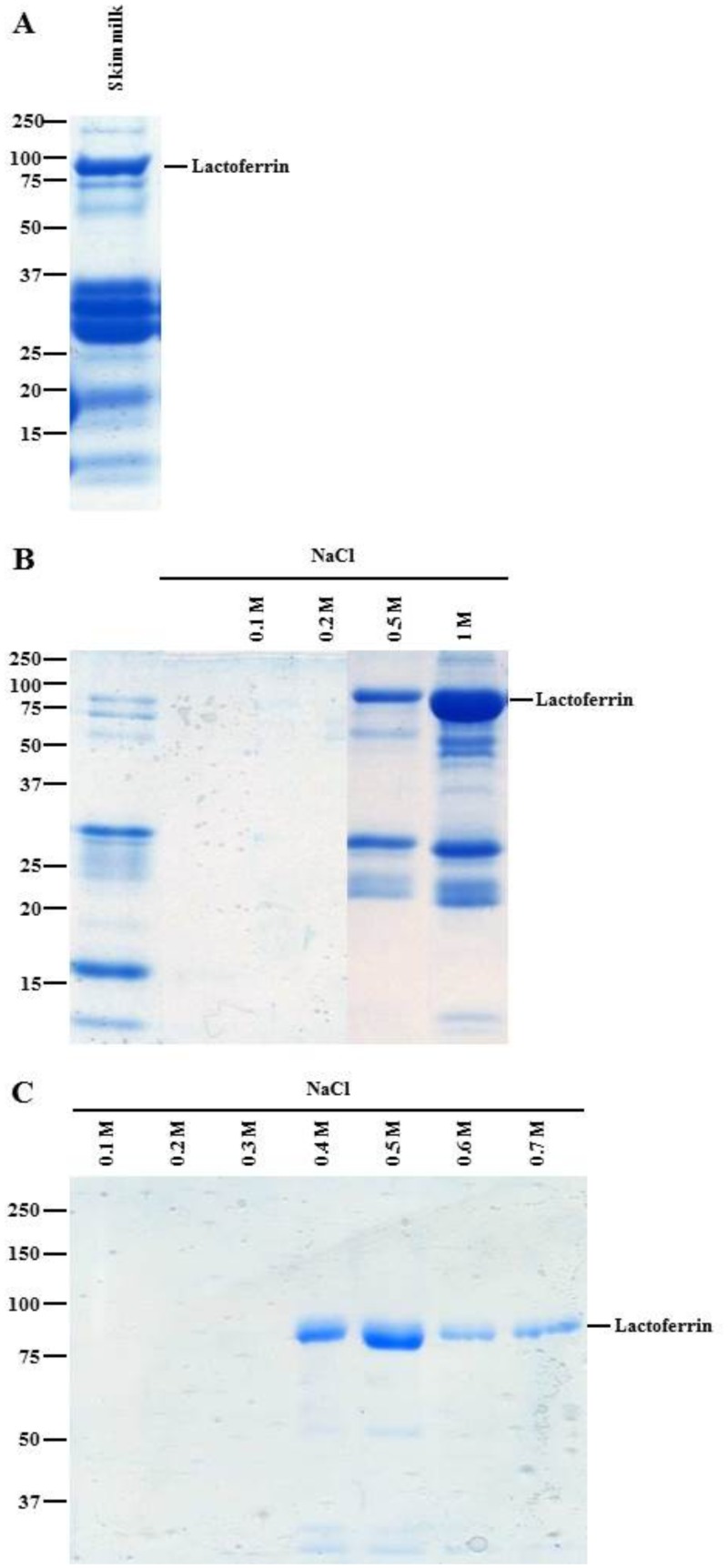
Recombinant human lactoferrin purification. (A) Skim milk protein profile analyzed by 12% SDS-PAGE. (B) Elution of rhLF purification by heparin Sepharose. (C) Elution of rhLF purification by ion-exchange chromatography.

Lipids were removed from milk by centrifugation and the caseins were eliminated by decreasing the pH 4.6 (isoelectric point of caseins). After heparin-Sepharose chromatography, eluted fractions were analyzed by SDS-PAGE (Fig 1A and 1B). SDS-PAGE of transgenic milk fractions showed a band around 80 kDa in the 0.5 M and 1 M NaCl fractions that corresponded to the molecular weight of LF. Previous analysis by MS confirmed the presence of LF in this band [30]. Caseins were present between 15 kDa and 37 kDa in skim milk. Acid precipitation was not sufficient to remove all caseins. Similar results were obtained for bLF (data not shown). To further improve the purity of the LF-enriched fraction, cation-exchange chromatography was performed to remove contaminants, including caseins. Some contaminants were eluted during the washing step with the running buffer, and LF was eluted by a stepwise elution using NaCl concentrations ranging from 0.1 M to 0.7 M. The majority of rhLF was present in the 0.5 M eluted fraction with low contamination (Fig 1C). This fraction had the level of purity necessary to perform analytical MS. Similar results were obtained for bLF (data not shown).

### Comparison of *N-*glycan composition

**Fig 2:**
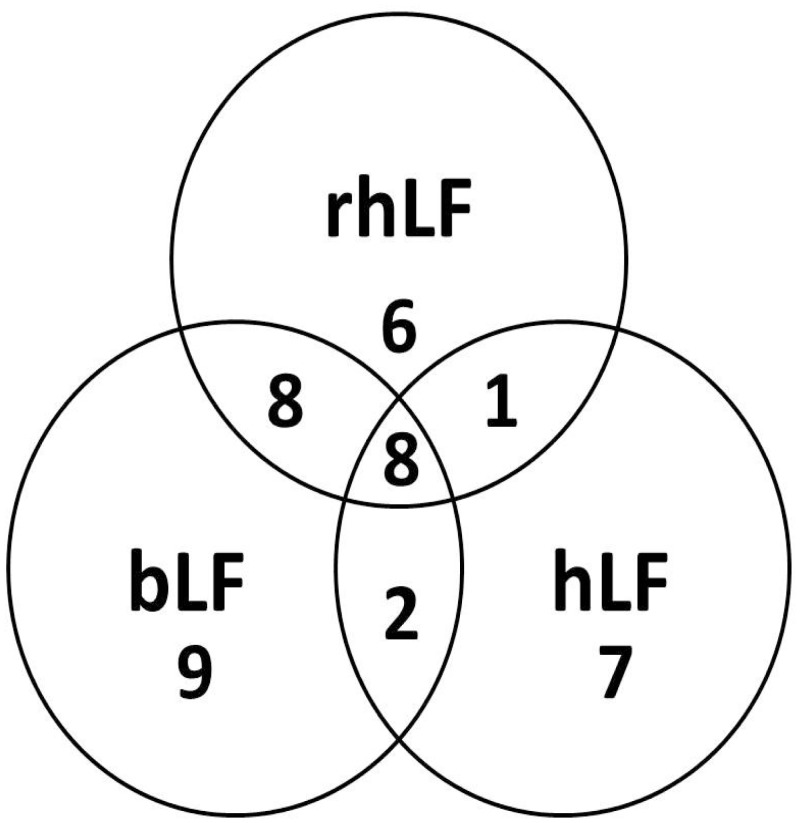
Comparison of bLF, rhLF and hLF *N-*glycan compositions.

The detailed compositions of *N-*glycans for bLF, rhLF and hLF are shown in S1, S2 and S3 Tables. PNGase F digestion resulted in 27, 23 and 18 different glycan compositions for bLF, rhLF and hLF, respectively (Table 1). Similar high mannose *N-*glycans were released from each LF, whereas the neutral non-fucosylated and fucosylated complex/hybrid *N-*glycan structures were different. rhLF contained the highest number of neutral non-fucosylated and fucosylated complex/hybrid glycans, with 7 compositions for both classes. rhLF contained 6 mono and 1 bi-fucosylated glycans, whereas bLF contained only 4 mono-fucosylated glycans and hLF had 3 mono-, 1 bi- and 1 tri-fucosylated *N-*glycans. rhLF and hLF showed similar NeuAc content, 5 and 6, respectively, and bLF had the highest sialylated glycan content, with 10 different compositions. It was also demonstrated that 2 NeuGc glycans were detected from bLF, whereas rhLF and hLF did not contain any NeuGc. Fig 2 compares *N-*glycan compositions of bLF, rhLF and hLF. Among the oligosaccharides identified, 8 *N-*glycan compositions were common among the three LFs. rhLF had more *N-*glycans in common with bLF than with hLF. bLF and rhLF had 16 glycans in common, whereas bLF and hLF shared 10 glycans. Among the 9 glycans in common between rhLF and hLF, only 1 uniquely belonged to these 2 LF samples. There were 9, 6 and 7 different glycan compositions unique for bLF, rhLF and hLF, respectively.

**Table 1:** *N-*glycan compositions of bLF, rhLF and hLF.

	bLF	rhLF	hLF
***N-*glycan compositions**	27	23	18
**High mannose**	5	4	5
**Neutral-non-fucosylated complex/hybrid**	6	7	2
**Neutral fucosylated complex/hybrid**	4	7	5
**Mono**	4	6	3
**Bi**	0	1	1
**Tri**	0	0	1
**NeuAc**	10	5	6
**NeuGc**	2	0	0

### Relative abundance of released glycans

**Fig 3:**
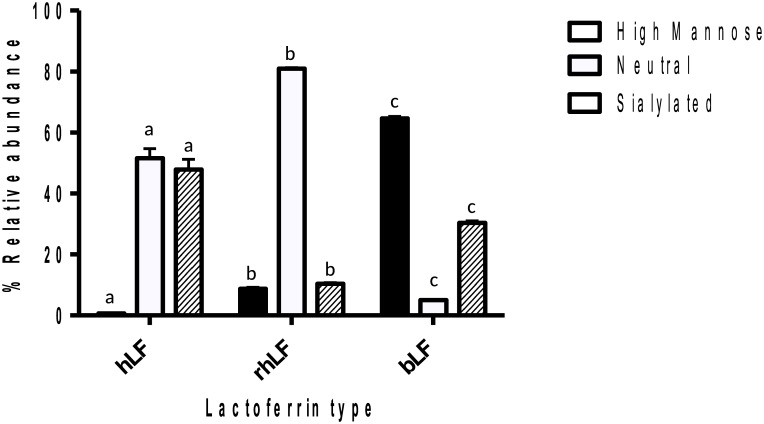
High mannose, sialylated and neutral complex/hybrid *N*-glycans released from hLF, rhLF and bLF by PNGase F. Tukey’s test was used to indicate significant differences (p<0.05) between groups. Same letters indicate no significant difference.

**Fig 4:**
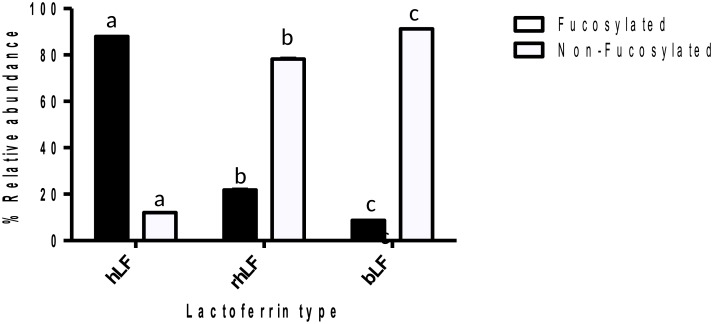
Fucosylated and non-fucosylated *N*-glycans released from hLF, rhLF and bLF by PNGase F. Tukey’s test was used to indicate significant differences (p<0.05) between groups. Same letters indicate no significant difference.

Relative abundances of released *N-*glycans of LF were compared. Fig 3 shows the relative quantitative distribution of high mannose, neutral complex/hybrid and sialylated complex/hybrid glycans, and Fig 4 shows fucosylated and non-fucosylated glycans. The results revealed that the relative abundance of each glycan type varied significantly for each LF. bLF was characterized by a high content of high mannose glycans (65%). The neutral complex/hybrid *N-*glycans represented only 4% of bLF *N-*glycans, and this class was significantly higher in hLF and rhLF, at 52% and 81%, respectively. rhLF sialylation (11%) was significantly lower than that for hLF and bLF (48% and 31%, respectively). The majority of glycans released from hLF were fucosylated (88%), whereas bLF and rhLF were 9% and 20% fucosylated, respectively (Fig 4).

### Comparison of *N*-glycan diversity of bLF, hLF and rhLF

**Fig 5:**
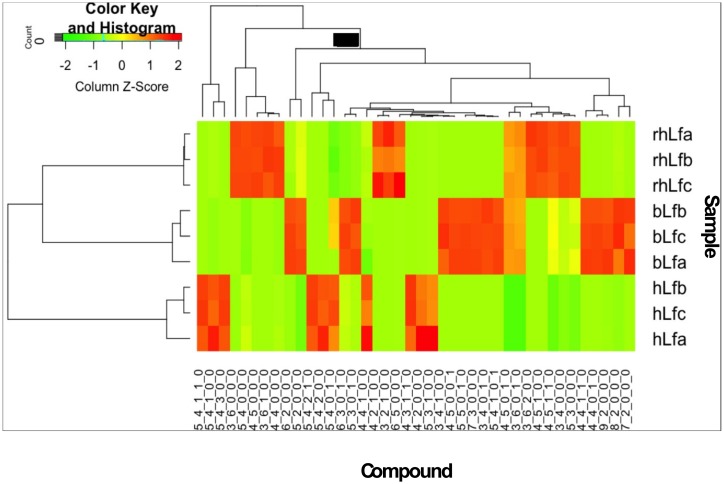
Heatmap of compound abundances associated with lactoferrin from different sources. Compound relative abundances were standardized (Z score, shown in legend) prior to unsupervised hierarchical clustering of samples (rows). Compound identity is noted below each column.

Application of bovine and human *N-*glycan libraries as a mass filter in Find by Molecular Feature with Mass Hunter Qualitative Analysis software identified 27, 23 and 18 *N-*glycan compositions for bLF, rhLF and hLF, respectively. From these compositions, 71, 49 and 66 compounds resulted from the separation of structural and/or linkage isomers or anomers for bLF, rhLF and hLF, respectively. Relative abundance of each *N-*glycan and comparison for each LF is shown in Fig 5. According to the heatmap, some *N-*glycans were unique for one lactoferrin sample and some others were mutual with various abundances. For example, compounds 4_5_1_0_0 and 3_6_2_0_0 are found in each lactoferrin but highly present in rhLF. 4_5_0_1_0 and 3_6_0_1_0 were present equally in rhLF and bLF but they were not detected in hLF. On the other hand, 5_4_0_0_0, 4_5_0_0_1 and 4_4_1_0_0 were unique *N-*glycans for rhLF, bLF and hLF, respectively.

**Fig 6:**
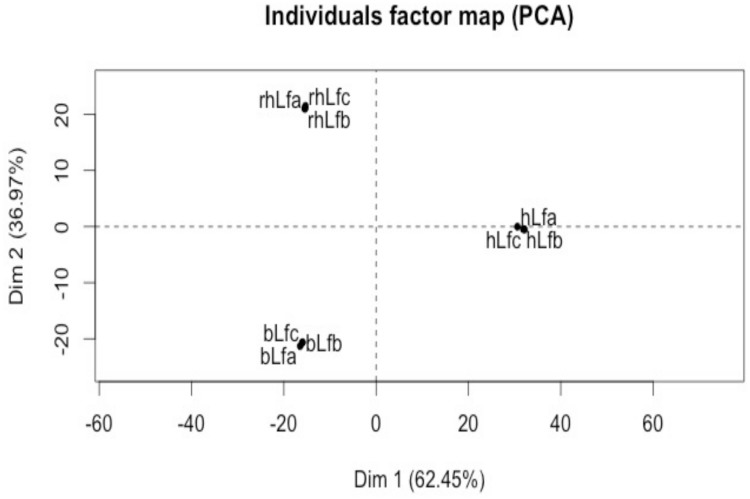
PCA plot of composition of released *N-*glycans of human lactoferrin (hLF), recombinant human lactoferrin (rhLfa) and bovine lactoferrin (bLf). Of the variance, 62.45% was explained by the first principal component and 36.97% was explained by the second principal component.

Moreover, the heatmap suggested that rhLF and bLF had more similarities than hLF based on the *N-*glycan diversity and their abundances. Although hLF shared 10 mutual *N-*glycans with bLF and 9 *N-*glycans with rhLF, bLF and rhLF shared a total of 16 *N*-glycans that were present in high abundance in these samples. In addition to the heatmap, a PCA plot (Fig 6) also shows that the variance between rhLF and bLF was less than between rhLF and hLF and hLF and bLF.

**Fig 7:**
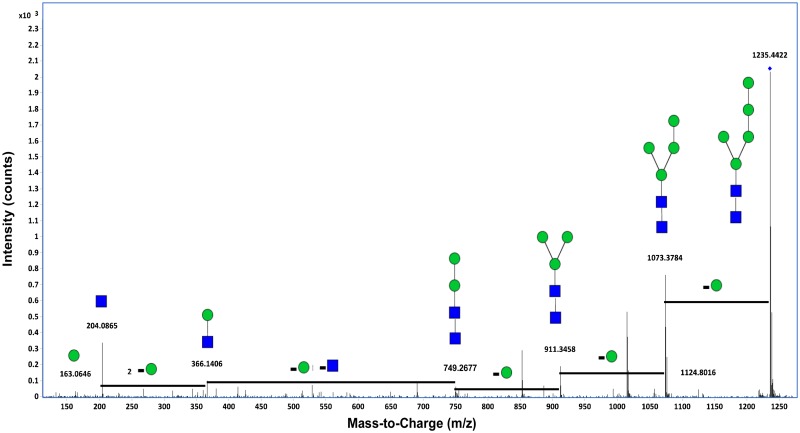
Deconvoluted tandem spectrum of the neutral N-glycan 5Hex2HexNAc from recombinant human lactoferrin. This glycan corresponds to 1235.44 m/z. Green circles and blue squares represent mannose and HexNAc residues, respectively.

**Fig 8:**
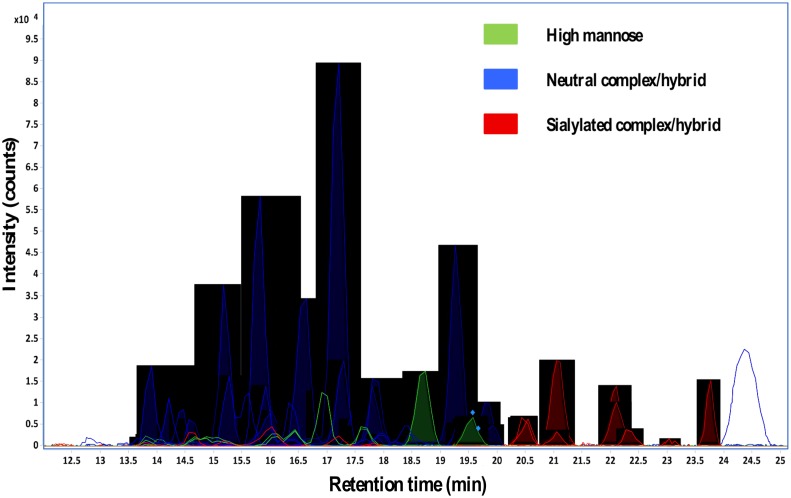
Extracted compound chromatograms (ECCs) of N-glycans from recombinant human lactoferrin.

A tandem spectrum of a neutral N-glycan and extracted compound chromatograms of N-glycans from recombinant human lactoferrins are shown in Figs 7 and 8, respectively.

## Discussion

Glycosylation of rhLF expressed in plants has been widely studied [31, 32], however, there is little information available for glycosylation patterns of rhLF expressed in the milk of transgenic cows. The objective of this study was to characterize the *N-*glycosylation pattern of rhLF, expressed in transgenic cows, using nanoLC-Chip-Q-TOF that allowed excellent separation performance and high mass accuracy.

After *N-*glycan release, a comparison study among hLF, bLF and rhLF *N-*glycans was performed. The analytical platform of nano-LC-Chip-Q-TOF MS enabled comprehensive profiling of *N-*glycans of hLF, bLF and rhLF and revealed heterogeneity of *N-*glycans with sialylated and fucosylated structures on rhLF. Although a few studies have reported the production of rhLF in transgenic cows, they focused on the monosaccharide composition of rhLF, and only few data are available on the *N-*glycosylation pattern of rhLF [33–35]. One of the first studies that compared the *N-*glycosylation pattern of rhLF expressed in transgenic cattle and hLF by MS was performed by Yu et al. [34]. They demonstrated that *N-*glycans from rhLF are mostly high mannose, hybrid and complex-type structures with less NeuAc and fucose, contrary to hLF that contains highly sialylated and fucosylated complex structures. Van Berkel et al. [36] showed that hLF contains complex-type glycans, and that rhLF produced in transgenic cows has more oligomannose and hybrid type glycans [33]. Similar to the study performed by Yu et al. [34], they show less sialylated and fucosylated glycans in rhLF than in hLF. Our results also showed that rhLF has fewer numbers of sialylated *N-*glycans than bLF and hLF. However, we demonstrated significantly higher fucosylation on rhLF (6 mono- and 1 bi-) than on bLF.

Our results showed that the *N-*glycome of rhLF is more similar to bLF than to hLF. According to previous studies [37], it is now widely known that fucosylation and sialylation make a difference between the bovine and human milk *N-*glycome. The bovine milk *N-*glycome contains more mannose and sialylated glycans than does the human milk *N-*glycome. Based on this information, because rhLF glycans were synthesized in bovine cells, we suggest that the differences in glycosylation patterns between hLF and rhLF may be the result of glycosyltranferases/glycosidases in bovine mammary epithelial cells. rhLF also contains unique *N-*glycans that are not identified on hLF or bLF. These glycans may have unique health-improving functions.

Although there is no evidence that different glycosylation patterns change LF activity, hLF and bLF might exhibit different functions, considering the multifunctional roles of glycans. Only a few papers report a role of glycosylation in protein function by modifying the structural conformation of the protein and consequently its biological activity [38, 39], or by interfering with pathogen adhesion to intestinal epithelial cells [40]. These possible actions strengthen the idea that glycosylation can be involved in protecting the host against microbial and viral attacks. Functional activities of rhLF were previously tested, and some anti-bacterial activities and iron binding of rhLF were similar to those of hLF [33, 41].

## Conclusion

Interest in LF is related to its wide range of biological properties that make it a major component for infant development. However, the mechanisms involved have not been well elucidated, especially the role that is played by glycan patterns on biological functions of LF. The results of our study could enable future investigations of the effects of glycosylation on LF properties.

The methodology employed in this study allowed isolation of a protein from a complex mixture, followed by the identification of *N-*glycans with high resolution and an isomer-level separation. *N-*glycan compositions released from commercial enzyme PNGase F were identified and compared among three LFs (bLF, hLF and rhLF). The results revealed a high diversity of *N-*glycan structures, including fucosylated and sialylated complex glycans, that may have bioactive potential. This study also increased the knowledge on rhLF and showed that it is a good substitute of bLF in bovine-based food. Transgenic cow’s milk that contain 1.2 g/L of rhLF could also be a good substrate for another novel enzyme called Endo-β-N-acetylglucosaminidase (EndoBI-1) of proteins [42–45]. Releasing higher amounts of lactoferrin’s glycans together with the un-glycosylated protein bone in its native (non-denatured) state will help understand the real contributions of *N-*glycans to lactoferrin’s functional activities.

LF supplementation has already many applications in food and non-food products around the world and its market continues to grow. The synthesis of rhLF *via* transgenic cows appears as a suitable production method that provides a large amount of rhLF with high homology with hLF. Numerous studies will be necessary to highlight the efficacy and safety of rhLF as a food additive or supplement.

## Supporting information

S1 Table Details of released rhLF *N*-glycans: Neutral mass and monosaccharide composition. HexNAc, *N*-acetylglucosamine; NeuAc, *N*-acetylneuraminic acid; NeuGc, *N*-glycolylneuraminic acid.
(DOCX)

S2 Table Details of released hLF *N*-glycans: Neutral mass and monosaccharide composition. HexNAc, *N*-acetylglucosamine; NeuAc, *N*-acetylneuraminic acid; NeuGc, *N*-glycolylneuraminic acid.
(DOCX)

S3 Table Details of released bLF *N*-glycans: Neutral mass and monosaccharide composition. HexNAc, *N*-acetylglucosamine; NeuAc, *N*-acetylneuraminic acid, NeuGc, *N*-glycolylneuraminic acid.
(DOCX)

## Abbreviations

bLF: bovine lactoferrin
Fuc: fucose
Hex: hexose
HexNAc: *N*-acetylglucosamine
hLF: human lactoferrin
LF: lactoferrin
MS: mass spectrometry
NeuAc: *N*-acetylneuraminic acid
NeuGc: *N*-glycolylneuraminic acid
*N-*glycan: *N*-linked glycan
PGC: porous graphitized carbon
rhLF: recombinant human lactoferrin
