# Supplementary material for: Characterization of recombinant human lactoferrin N-glycans expressed in the milk of transgenic cows
Source: PLoS One. 2017 Feb 7;12(2):e0171477. doi: 10.1371/journal.pone.0171477 (PMC5295716; doi:10.1371/journal.pone.0171477)
Supplement: S1 Table — HexNAc, N-acetylglucosamine; NeuAc, N-acetylneuraminic acid; NeuGc, N-glycolylneuraminic acid. (DOCX) [file pone.0171477.s001.docx]

**Table S1.** Details of released rhLF *N*-glycans: neutral mass and monosaccharide composition. HexNAc, *N*-acetylglucosamine; NeuAc, *N*-acetylneuraminic acid; NeuGc, *N*-glycolylneuraminic acid.

| **Hexose** | **HexNAc** | **Fucose** | **NeuAc** | **NeuGc** | **Mass** |
| --- | --- | --- | --- | --- | --- |
| 3 | 2 | 1 | 0 | 0 | 1056.39 |
| 4 | 2 | 1 | 0 | 0 | 1218.44 |
| 5 | 2 | 0 | 0 | 0 | 1234.43 |
| 3 | 4 | 0 | 0 | 0 | 1316.49 |
| 6 | 2 | 0 | 0 | 0 | 1396.49 |
| 5 | 3 | 0 | 0 | 0 | 1437.51 |
| 4 | 4 | 0 | 0 | 0 | 1478.54 |
| 4 | 4 | 1 | 0 | 0 | 1624.60 |
| 5 | 4 | 0 | 0 | 0 | 1640.59 |
| 4 | 5 | 0 | 0 | 0 | 1681.62 |
| 8 | 2 | 0 | 0 | 0 | 1720.60 |
| 3 | 6 | 0 | 0 | 0 | 1722.65 |
| 5 | 4 | 1 | 0 | 0 | 1786.65 |
| 4 | 5 | 1 | 0 | 0 | 1827.68 |
| 3 | 6 | 1 | 0 | 0 | 1868.71 |
| 9 | 2 | 0 | 0 | 0 | 1882.64 |
| 5 | 4 | 0 | 1 | 0 | 1931.69 |
| 4 | 5 | 0 | 1 | 0 | 1972.72 |
| 6 | 5 | 0 | 0 | 0 | 2005.73 |
| 3 | 6 | 0 | 1 | 0 | 2013.74 |
| 3 | 6 | 2 | 0 | 0 | 2014.74 |
| 5 | 4 | 1 | 1 | 0 | 2077.75 |
| 4 | 5 | 1 | 1 | 0 | 2118.77 |
