# Supplementary material for: Characterization of recombinant human lactoferrin N-glycans expressed in the milk of transgenic cows
Source: PLoS One. 2017 Feb 7;12(2):e0171477. doi: 10.1371/journal.pone.0171477 (PMC5295716; doi:10.1371/journal.pone.0171477)
Supplement: S3 Table — HexNAc, N-acetylglucosamine; NeuAc, N-acetylneuraminic acid, NeuGc, N-glycolylneuraminic acid. (DOCX) [file pone.0171477.s003.docx]

**Table S3.** Details of released bLF *N*-glycans: neutral mass and monosaccharide composition. HexNAc, *N*-acetylglucosamine; NeuAc, *N*-acetylneuraminic acid, NeuGc, *N*-glycolylneuraminic acid.

| **Hexose** | **HexNAc** | **Fucose** | **NeuAc** | **NeuGc** | **Mass** |
| --- | --- | --- | --- | --- | --- |
| 5 | 2 | 0 | 0 | 0 | 1234.43 |
| 3 | 4 | 0 | 0 | 0 | 1316.49 |
| 6 | 2 | 0 | 0 | 0 | 1396.49 |
| 5 | 3 | 0 | 0 | 0 | 1437.51 |
| 3 | 4 | 1 | 0 | 0 | 1462.55 |
| 7 | 2 | 0 | 0 | 0 | 1558.54 |
| 3 | 4 | 0 | 1 | 0 | 1607.58 |
| 4 | 4 | 1 | 0 | 0 | 1624.60 |
| 5 | 4 | 0 | 0 | 0 | 1640.59 |
| 8 | 2 | 0 | 0 | 0 | 1720.59 |
| 3 | 6 | 0 | 0 | 0 | 1722.64 |
| 5 | 3 | 0 | 1 | 0 | 1728.61 |
| 7 | 3 | 0 | 0 | 0 | 1761.62 |
| 4 | 4 | 0 | 1 | 0 | 1769.64 |
| 5 | 4 | 1 | 0 | 0 | 1786.65 |
| 3 | 6 | 1 | 0 | 0 | 1868.70 |
| 9 | 2 | 0 | 0 | 0 | 1882.64 |
| 6 | 3 | 0 | 1 | 0 | 1890.66 |
| 4 | 4 | 1 | 1 | 0 | 1915.69 |
| 5 | 4 | 0 | 1 | 0 | 1931.69 |
| 4 | 5 | 0 | 1 | 0 | 1972.71 |
| 4 | 5 | 0 | 0 | 1 | 1988.71 |
| 3 | 6 | 0 | 1 | 0 | 2013.74 |
| 5 | 4 | 1 | 1 | 0 | 2077.75 |
| 5 | 4 | 1 | 0 | 1 | 2093.74 |
| 4 | 5 | 1 | 1 | 0 | 2118.78 |
| 5 | 5 | 0 | 1 | 0 | 2134.77 |
